# Evaluation means making CQI collaborative (E = MC^2^): Lessons learned from implementing a new continuous quality improvement process at the Einstein-Montefiore CTSA hub

**DOI:** 10.1017/cts.2025.10136

**Published:** 2025-08-27

**Authors:** Ariel Y. Fishman, David Lounsbury, Claudia Lechuga, Jessica Kahn, Mimi Y. Kim, Marla Keller

**Affiliations:** Harold and Muriel Block Institute for Clinical and Translational Research, Albert Einstein College of Medicine and Montefiore Medical Center, Bronx, NY, USA

**Keywords:** Evaluation, continuous quality Improvement, reflexive inquiry, strategic management, NCATS

## Abstract

The NIH’s Clinical and Translational Science Award (CTSA) program has placed greater emphasis on Continuous Quality Improvement (CQI) in recent years. Our institution’s CTSA-supported Institute for Clinical and Translational Research (ICTR) implemented a novel CQI process in response. This manuscript shares lessons learned from our implementation, reflecting a paradigm shift from managing an “evaluation program” to creating a process whose central goal is CQI. Our objective is to share these reflections to support other CTSA hubs’ efforts to successfully implement CQI programs. Key elements of our implementation included (1) establishing a shared understanding about CQI’s purpose; (2) leveraging a centralized management approach while addressing barriers to implementation; and (3) creating structures that foster collaboration. The CQI framework we chose, FACE (Focus, Analyze, Change, Evaluate), enabled us not only to improve the activities of ICTR modules but also, over time, to refine the CQI process itself. Through regular convenings of module leaders, the ICTR has sought to cultivate a culture of CQI as a dynamic, participatory process that supports mutual learning and collective problem-solving.

## Introduction

In 2021, the National Institutes of Health’s (NIH’s) National Center for Advancing Translational Sciences (NCATS) issued a new Funding Opportunity Announcement (FOA) for its Clinical and Translational Science Award (CTSA) Program, which serves to advance its mission of translating research discoveries into improved patient care and population health. In a change from prior announcements, this new FOA required that CTSA hub applicants establish “a strong Continuous Quality Improvement (CQI) program, which is an ongoing cycle of collecting data and using it to make decisions to gradually improve program processes”[1]. The new FOA did not guide or obligate hubs to utilize a particular CQI approach; indeed, more recent versions of the FOA note that “the methods… chosen to perform this CQI are not specified… to allow flexibility for the applicants to choose the method(s) that are most appropriate for their proposed hub’s needs”[2]. Rather, the FOA’s requirements simply included a section entitled, “Continuous Quality Improvement and Program Evaluation,” which asked applicants to describe their “plans for CQI, monitoring, and how interventions will be implemented when indicated” as well as “plans to collect data to evaluate the impact of the CTSA award”[1].

This requirement represented an evolutionary change from earlier CTSA FOA expectations, which centered primarily around the obligations of program evaluation[3,4]. These earlier approaches held that collecting standardized metrics would naturally motivate grantees to improve in areas where performance fell short of a benchmark. Under the former paradigm, data collection and monitoring efforts were centered on showing that grant-funded programs were “well implemented, efficiently managed, and demonstrably effective”[5]. Manifestations of this approach included Research Performance Progress Reports (RPPRs) containing standardized data tables, along with the Common Metrics Initiative, established by NCATS to support “a formalized and standardized evaluation process”[6,7] that would implicitly lead hubs to focus on improvement.

The CTSA hub at Albert Einstein College of Medicine, and its clinical partner, Montefiore Health System, developed a process to address the newly required CQI component of the FOA, listed under Element B: Strategic Management[1]. The Einstein-Montefiore hub – known as the Harold and Muriel Block Institute for Clinical and Translational Research (ICTR) – engaged in formative, qualitative collaboration among the leaders of our component modules to design a new CQI program that reduced emphasis on collecting standardized metrics used – at best – solely for impact evaluation purposes, and increased emphasis on a process of defining metrics internally that were more directly aligned with module-specific goals. NCATS awarded a UM1 grant to the ICTR in March 2023.

In this manuscript, we reflect on lessons learned from our implementation approach. These include (1) developing a shared understanding and language for what CQI is as well as its scope and strategic value; (2) building the case for centralized CQI management; (3) providing appropriate structures to both cross-pollinate ideas and foster trust among module leaders and the CQI team; and (4) engaging in efforts to improve the CQI process itself. Throughout these lessons, we noted certain core principles crucial to our work, including visible support from ICTR leadership and collaborative stakeholder empowerment and engagement. Our objective is to share a set of reflective observations that may be useful to other CTSA hubs as they develop and implement their respective CQI programs.

### Methods

#### Reflexive inquiry

We applied tenets of reflexive inquiry (RI) to analyze what we learned in developing our hub’s CQI process. In RI, investigators critically examine their own role, assumptions, values, and influence throughout the research process. Reflexivity helps investigators identify and understand how their relationship to a given topic and to the sources of qualitative data in a research project shapes a study’s purpose, context questions, methods, and validity[8]. RI is often employed by qualitative researchers who have the role of observing or facilitating a given intervention, and helps to assess biases in their own perspective while formalizing an intuitive understanding of the processes and outcomes of that intervention.

Our CQI team holds a wide range of professional evaluation expertise, with extensive backgrounds in clinical and translational research, community engagement, implementation science, strategic management, education, biostatistics, and informatics. The team includes three of this manuscript’s co-authors: an organizational behavior specialist who conducts evaluations for higher education accreditation (A. Fishman); a community psychologist with extensive evaluation experience (D. Lounsbury); and a Director of Evaluation and Tracking efforts who also manages the ICTR’s participation in the CTSA’s Common Metrics program (C. Lechuga).

We applied RI to consider the benefits, challenges, and outcomes of our CQI process through periodic meetings among the CQI team, between the team and ICTR leadership, and by reviewing feedback collected in post-CQI session evaluation surveys. Consistent with the notion of “bracketing” a strategy supporting RI[9], our weekly CQI team meetings included dedicated time to reflect on the perceived effectiveness of our facilitation of the CQI model. During these meetings, each CQI team member discussed their understanding of how best to shape the CQI cycle for the ICTR modules to which they were assigned, while the other CQI team members offered feedback and suggestions. The present manuscript reflects the cumulative insights gained through this reflective process. Our analysis centers on our perspective as members of the CQI team and is informed by feedback collected from module leaders through formal surveys and informal conversations conducted over the three years since the CQI program’s initial implementation.

As our implementation unfolded, we encountered certain hurdles. Below, we describe the strategies we used to address them. While we aim to accurately represent the views of colleagues across the ICTR, in some cases, we describe them in stylized or general terms to illustrate broader patterns we observed, even if they were not explicitly expressed as positions held by any particular individual.

#### Our CQI process

.We describe the steps used to implement our CQI process in a prior publication[10] and summarize them again in Table 1. First, our ICTR’s principal investigators (PIs) established the CQI team as a dedicated set of professionals tasked with launching a new approach to foster engagement with CQI. Team members were formally allocated budgetary time/effort, and, in their leadership capacity, the PIs made regular affirmations of their engagement with CQI. Next, the ICTR’s module leaders, each responsible for advancing a specific aim such as workforce development or community engagement, created LMs that articulated module-level goals, activities, intended impact, and metrics. Module leaders were charged with establishing mechanisms for recording and retaining data related to metrics of interest. We adapted FACE (Focus, Analyze, Change, Evaluate) as our model for improvement, and took the position that the success of our CQI program depended less on the choice of model – whether Six Sigma, Plan-Do-Study-Act, Lean Management, or Root Cause Analysis – and more on disciplined adherence to schedule. We launched improvement projects with each ICTR module on a staggered basis to (1) ramp up and sustain momentum for ongoing CQI, (2) utilize the limited capacity of the CQI team efficiently to manage concurrent projects, and (3) foster collaboration across modules. We convened with module leaders across CQI project phases every four weeks (later changed to every six weeks) to share updates, solicit feedback, and disseminate insights gained from their work. Finally, we actively collected feedback on the CQI process itself, iteratively refining it to enhance its effectiveness and maintain stakeholder engagement.

**Table 1.** Six steps for implementing a continuous quality improvement program^[10]^

***1. Establish a foundation:*** Commit to engage in CQI through clear support from leadership; a shared desire to improve through collaboration, transparency, and discipline; and sufficient time, personnel, and financial resources to succeed.
***2. Define goals:*** Utilize tools like Logic Models (LMs) to articulate desired impact, measurable goals, and activities that advance those goals. Iteratively revise the LMs to ensure alignment across the organization.
***3. Collect relevant metrics:*** Establish reliable, adaptable, and accessible mechanisms to collect and store data to assess progress toward goals and identify potential areas for improvement.
***4. Apply a Quality Improvement (QI) framework:*** Using a structured framework such as FACE (Focus/Analyze/Change/Evaluate), identify and conduct small-scale improvement projects that advance progress toward long-term goals in ways that are measurable and that can inform future work.
***5. Continuously engage and cross-pollinate:*** Maintain momentum and manage resources by staggering multiple concurrent QI projects; convene leaders at different project phases to share progress, collect feedback, and disseminate lessons learned.
***6. Assess and adjust:*** Ensure that the CQI process itself can be altered using feedback from key stakeholders actively collected across repeated CQI cycles.

#### Principles and conceptual framework

Our CQI implementation intentionally aligns with certain core principles of translational science[11]. First, in the same way that community engagement can improve the quality of clinical and translational research, we held that ***engaging stakeholders*** within our CTSA hub would deepen their investment in CQI[12–14]. Accordingly, our CQI process empowered module leaders to independently select CQI projects, rather than requiring them to define their work solely based on performance on externally defined metrics. Leaders selected projects by analyzing internally defined metrics or drawing on their intuitions and tacit knowledge and were encouraged to consult – and, if necessary, revise – their LMs to identify appropriate improvement opportunities. Leaders were encouraged, but not required, to engage with the CQI team to select a project, calibrate its scope, or leverage the team’s familiarity with related projects conducted by other modules. Second, noting that ***creativity and innovation*** in science requires an environment in which failure creates learning and improvement opportunities[15,16], we prioritized building trust among stakeholders through collaboration. Consistent with best practice in collaborative program evaluation, we worked to establish an atmosphere of open, honest communication where in-progress and incomplete work were shared as a norm[17]. Third, noting that ***efficiencies*** in project management and organization drive scientific progress[18,19], we designed our approach to foster small wins to advance incremental progress toward longer-term goals. Doing so enabled the ICTR and its modules to rapidly adjust our activities based on timely data about whether they were succeeding at achieving measurable goals.

### Results

#### Developing a shared definition of CQI

In the early writing stages of our grant renewal, we came to recognize that our colleagues held many different conceptual understandings of CQI, including its definition, axiomatic frameworks, purpose, and presumed output. Conceptions of CQI reflected a variety of experiences with metric- and compliance-driven reporting processes (such as RPPRs), overlapping language used in contexts such as accreditation, limited exposure to improvement models, or assumed norms in which metrics were seen solely as a tool for enforcing accountability rather than facilitating improvement. Some colleagues had experience with improvement frameworks such as PDSA, LEAN, or Six Sigma, leading to heterogeneous perceptions about CQI’s purpose, scope, and strategic value. While each experience and understanding has merit, we strived to use a shared definition of CQI to minimize confusion and bolster effective collaboration. We set aside time to review our CQI framework and intended process in our early meetings with leaders of each module and with the ICTR leadership team to ensure alignment and shared understanding. At ongoing convenings, we presented metaphors to explain the intended role of CQI; for example, we asked module leaders to imagine themselves as ship captains, with CQI helping to adjust the rudder’s ability to steer that ship toward its desired destination. We also developed a brief guidance document that we sent to module leaders prior to initiating each CQI project.

Over the course of our implementation, we observed or encountered five general *a priori* assumptions about CQI. Our organization of these assumptions emerged during the CQI team’s RI process. These observations emerged from discussions of post-project evaluations, analysis of open-ended survey responses (see section below entitled, “Conducting CQI of the CQI process”), and ongoing observation as participants throughout successive cycles of CQI projects. We outline these five assumptions below, examine the challenges they posed to implementation, and describe our efforts to mitigate them.

#### Assumption 1: CQI is documentation

*CQI is about collecting documentation to fulfill mandates created by external stakeholders – such as granting agencies, accrediting bodies, or advisory groups – rather than generating substantive change*. To counter this idea, we emphasized a vision of CQI primarily as a mechanism for achieving short- and long-term goals, and that collecting documentation associated with process improvement would be ancillary. We acknowledged that documentation of CQI efforts could satisfy compliance requirements, but we guided module leaders to imagine how an effective CQI project might be executed if no documentation were required.

#### Assumption 2: CQI is an audit

*CQI begins and ends by providing data on predefined metrics and is not necessary in areas where performance is perceived as adequate; improvement beyond an already-satisfactory threshold is not noteworthy.* Here, we first emphasized that the central aim of CQI is to maximize successful goal achievement regardless of current performance levels. Building on this emphasis, we encouraged module leaders to consider developing a hypothesis for how achieve this improvement. Finally, we encouraged modules to collect and analyze data to test that particular hypothesis.

#### Assumption 3. CQI diverts resources/attention

*CQI projects are inherently resource intensive and make it difficult to give sufficient attention to operational activities directly associated with primary goals.* To mitigate this notion, we reinforced a message that the CQI process should catalyze, rather than prevent, goal achievement. We emphasized the intentional alignment of CQI with day-to-day activities. Finally, we underscored that incremental interventions and adjustments to day-to-day activities could lead to substantial long-term improvement, much in the same way that taking time to sharpen the blade of a saw could affect its ability to cut wood[20], and accelerate progress toward a desired goal.

#### Assumption 4: CQI can be delegated

*CQI projects could be delegated to colleagues not in leadership roles.* Similar to the view of CQI as merely documentation, this perspective considered CQI as constrained to a data collection and reporting process. Confronted with such situations, the CQI team acknowledged that certain tasks like data collection can reasonably be delegated. At the same time, we emphasized areas where leadership involvement was particularly necessary for effective CQI, highlighting leaders’ critical role in project selection and change management.

#### Assumption 5: CQI is temporary support

*The CQI team functions to provide temporary assistance with operational activities.* We surmised that since a novel injection of surplus resources could indeed help module leaders advance achievement of their goals, the CQI team’s participation in component operations could mistakenly be framed as an “improvement activity.” In such cases, we clarified that the aim of CQI (and the role of the CQI team’s allocated time) was to help identify where changes to existing processes – *beyond* simply increasing resource allocation – could improve outcomes.

#### Centralizing CQI into strategic management

Even with a shared understanding of CQI’s purpose and shared frameworks in place, a key strategic decision was determining the extent to which CQI management should be centralized. In a decentralized model, module leaders would engage in CQI projects independently, allowing substantial flexibility in projects’ frequency, duration, intensity, and scheduling. We recognized that centralized models offer important benefits, including the ability to standardize processes, implement best practices, and ensure satisfactory execution of CQI. Our interpretation of the UM1 FOA, following the guidelines as stated[1], was that a centralized CQI model was preferable, given CQI’s placement as a component of the strategic management element. Importantly, we interpreted this approach to mean that, rather than having the CQI team determine the content of projects, its primary role was to coordinate and align modules with a unitary methodology across the ICTR.

We also acknowledged some of the costs of centralizing CQI. These costs included communicating CQI planning with module leaders and coordinating the scheduling availability of CQI team members with that of the modules with which they were engaged. For example, we intentionally avoided launching a CQI project during periods when a module faced a temporarily high workload, such as when key staff transitions were underway or around application deadlines.

To support the centralized model, the CQI team established an annual schedule for all modules’ CQI projects with a standardized project duration of about three months (later lengthened to five months based on module leader feedback). Three overlapping projects were scheduled to occur on a staggered basis. Modules with anticipated CQI projects of their own, and of appropriate intended duration, were invited to schedule their projects to coincide with the central CQI schedule, in the event they wanted to utilize the CQI team’s time and expertise as a resource. Module leaders seeking to advance projects lasting more than a few months were invited to consider subprojects with which the CQI team could be involved. Module leaders were also welcomed to engage in additional independent CQI projects, manifesting a culture of continuous improvement across the ICTR and the institution as a whole, but such additional independent projects could be completed without the coordinated participation of the CQI team.

The CQI team met with each module leader at the outset of the project period to help guide project selection, particularly to support modules choosing among a list of options or considering a project whose feasibility seemed risky. Module leaders were not obligated to involve the CQI team in day-to-day project management – we noted that the CQI team tended to be less involved in projects requiring technical knowledge or context familiarity unique to a specific module. In all cases, the minimum obligation required of each module leader was to execute a CQI project of appropriate size and substance, to communicate project status with the CQI team every few weeks, and to present the project’s progress to other modules at scheduled CQI meetings. The CQI team aimed to balance flexibility and engagement against the ICTR’s resource constraints and the challenges of managing a complex schedule.

Reluctance to participate in CQI’s centralization generally fell into one of three categories of mindsets, perhaps simplified as “We’re too busy,” “Performance is satisfactory,” or “We’re already doing CQI.” We acknowledged that these mindsets were likely shaped by day-to-day pressures of limited resources and competing priorities, as well as differing experiences with previous CQI efforts and perceptions of its utility.

For leaders expressing a lack of time or resource availability, the CQI team positioned its involvement as offering analytical support, expertise, new frameworks, and connections to peers tackling similar challenges. In these cases, the CQI team focused its efforts toward building partnership, trust, and enhancing module leaders’ ability to identify and address the root causes of their challenges.

For leaders who might have deferred CQI because they perceived current performance as adequate or satisfactory, the CQI team emphasized that even high-performing areas could achieve improvement. Changing circumstances such as new technologies, regulatory environments, institutional priorities, or resource availability could uncover new opportunities as well as produce valuable insights for other modules facing similar circumstances.

For leaders who indicated that they were already doing CQI, the CQI team adjusted the cadence and schedule of its support to align with efforts in progress. The CQI team emphasized the benefit of collaboration with other ICTR module leaders, using team meetings as opportunities to cross-pollinate insights, leverage collective expertise, and enhance the impact of CQI initiatives. Centralized participation also ensured that consistent CQI methodologies and frameworks were being applied across the ICTR.

Module leaders’ mindsets were more thoughtful, complex, and nuanced than the simplifications we presented above. No matter the mindset, however, the CQI team approached each leader as a partner, aiming to work as a trusted collaborator with which failures could be openly discussed. We ensured that the module leader, not the CQI team, received primary credit for identifying and executing on an improvement opportunity.

#### Building a collaborative culture by cross-pollinating ideas

In our CQI methodology, described elsewhere in greater detail[10], we convene meetings with all module leaders every four to six weeks. (Of note, our original process entailed that only the three presenting modules were invited to meetings, but we later expanded the meeting invitation list to non-presenting modules based on feedback we collected. See “Conducting CQI of the CQI process,” below.) In each of these meetings, one module leader presents plans for an upcoming CQI project, a second discusses a CQI project in progress, and the third reflects on lessons learned from a recently completed CQI project. Meeting participants, especially other module leaders, then provide feedback and contribute to each other’s initiatives. Sessions are enriched by the intentional juxtaposition of new, ongoing, and concluding projects discussed in the same session. At the start of these meetings, the CQI team reinforces our shared definition of CQI, emphasizes safe-space norms for sharing learnings from failure or underdeveloped ideas, and actively seeks to facilitate opportunities for modules to collaborate on projects. Over time, these convenings have helped demonstrate to stakeholders that CQI is a dynamic, participatory process that supports mutual learning, resource-sharing, and collective problem-solving.

The byproduct of these meetings has been a series of cross-pollinations that have fostered a culture in which collaboration is encouraged, experimentation is valued, and CQI is increasingly viewed as a shared responsibility across the ICTR rather than a siloed responsibility for each individual module or for the CQI team alone. Such discussions not only succeeded at cross-pollinating ideas but also created material efficiencies in our projects: For example, one leader expressed plans to administer a CQI survey, prompting another module leader to share plans for a similar survey and propose collaborating on a unified survey with separate sections to serve each module’s CQI needs.

Leaders might also outline a project that draws on expertise available outside their module. For example, the director of a training program presented a CQI project involving thematic analysis of past applications, and the director of the informatics module offered to expedite the analysis using a large language model or generative artificial intelligence tool. Similarly, a module teaching a course on research methods was able to offer its students as participants for another module’s focus group, creating a mutually beneficial opportunity that advanced both the students’ learning experience and the data collection needs for the focus group. These experiences manifest the NCATS core principle of Cross-Disciplinary Team Science, in which expertise from across different disciplines, fields, and professions produces substantive improvements[11,16].

#### Conducting CQI of the CQI process

At the conclusion of every CQI meeting, the CQI team administered a survey to all meeting participants to ensure that the meeting accomplished its intended purpose and to collect data that could be used to refine the CQI process itself. Survey questions include Likert-style agreement questions about whether CQI team members “encouraged our active participation in decision-making,” “made sure we accomplished our goals,” and “made good use of our time.” Over the three years since its launch, 89% of respondents indicated they “strongly agree” or “agree” with each of these statements.

The survey collects open-ended comments as well. To date, feedback from module leaders has been validating (“Love hearing ideas from everyone and problem solving as a group;” “Good to hear about others work and be able to give feedback or just know what is going on outside my core;” “Very supportive environment for everyone to provide constructive feedback” and “The input from the CQI team throughout this cycle was instrumental to improving”) but has also revealed meaningful improvement opportunities. For example, one comment stated, “Would be great if other leaders could join who are not presenting,” leading the CQI team to extend meeting invitations not only to three module leaders actively engaged in CQI projects but to the leaders of all ICTR modules and administrative leaders. A comment that “more time is needed between meetings to implement changes or observe outcomes of changes” led the CQI team to expand the duration between meetings from four weeks to six weeks. Establishing a transparent mechanism for the CQI team to engage in an improvement process helped, as well, to foster a culture in which continuous feedback and collaboration are integral to achieving sustained progress for all.

## Discussion

Our Reflective Inquiry indicates that our CQI program’s success emerged from four steps: (1) establishing a shared definition of CQI; (2) centralizing CQI into strategic management; (3) creating mechanisms to cross-pollinate ideas and foster trust among stakeholders; and (4) ensuring that the CQI process itself benefited from CQI. Collectively, each step was taken in collaboration and partnership between module leaders, ICTR leadership, and the CQI team, helping to establish and deepen a culture of trust, mutual agreement, and engagement. This alignment and investment toward trust became foundational to the success and sustainability of our CQI approach.

We do not claim to have perfected a unique formula for the success of our CQI process, nor do we assert that they apply only to CQI processes for CTSA hubs. Nonetheless, we observed that certain ingredients seemed to have played a consistent role. Chief among them has been the unwavering commitment of senior leadership, which the CQI team viewed as foundational. This commitment manifested not only through the inclusion of CQI representatives at leadership meetings and the requirement that all module leaders develop logic models (LMs) and engage in CQI efforts, but also in the grant-funded allocation of protected time for the CQI team to meet weekly and for module leaders to participate in project meetings every four to six weeks. Administrative support and discipline to schedule meetings has been particularly critical as well.

Above all else, the CQI team made every effort to maintain a collaborative atmosphere with module leaders, ensuring they felt supported in project selection and implementation. We continuously discussed with module leaders how they could integrate CQI into their core activities, making CQI a natural extension of their existing operational tasks rather than seeming like an additional workload. The CQI team deliberately empowered module leaders to select CQI projects, and trusted them to design projects that aligned with their module goals and the goals of the ICTR as a whole. Through this strategic tradeoff, we respected the preferences of module leaders rather than imposing a centralized perspective on what needed improvement, even as we maintained a centralized approach to facilitating the improvement process. Module leaders reported to us that our approach helped them gain familiarity with the CQI process and its purpose, recognizing how it served to advance the goals of the modules and the ICTR as a whole.

We acknowledge that, by their nature, the reflections presented in this manuscript reflect our collective experiences and shared interpretations developed through reflective inquiry rather than the explicit findings of a data analytic approach. Consequently, others’ experiences with CQI implementation may differ from what we described. Our experiences may also be a function of the specific institutional context in which our hub operates. Nonetheless, as other CTSA hubs begin to incorporate CQI as explicitly mandated by NIH granting agencies and other regulatory bodies, we hope that these reflections will provide valuable insights into the implementation of CQI initiatives at other institutions.

We suggest several potential directions for further research. Disseminating these methods more formally to other CTSA hubs could enhance collaborative efforts and standardize best practices. We aim to place particular focus on fostering cross-module collaborations more regularly and measuring progress toward a culture of continuous improvement. Additionally, understanding how CQI directly impacts translational science represents a significant opportunity to advance the CTSA’s mission and contribute to the broader field of translational research.
